# Pre-mRNA splicing and its regulation in microalgae and cyanobacteria

**DOI:** 10.1007/s44307-025-00087-3

**Published:** 2025-12-14

**Authors:** Sally Do, Yue Liu, Henry Huynh, Yinggao Liu, Wujiao Li, Mo-Xian Chen, Zhi-Yan Du

**Affiliations:** 1 Present Address: Department of Molecular Biosciences and Bioengineering, University of Hawaii at Manoa, Honolulu, HI 96822 USA; 2 Department of Laboratory Medicine, Shenzhen Children’s Hospital, Shenzhen, 518038 China; 3 Present Address: State Key Laboratory of Tree Genetics and Breeding, Key Laboratory of State Forestry and Grassland Administration On Subtropical Forest Biodiversity Conservation, the Southern Modern Forestry Collaborative Innovation Center, College of Life Sciences, Nanjing Forestry University, Nanjing, 210037 China; 4 State Key Laboratory of Wheat Improvement, College of Life Science, Shandong Agricultural University, Taian, 271018 Shandong China

**Keywords:** Alternative splicing, Bioreactor, Post-transcriptional regulation, Eukaryotes, Splicing machinery, Photosynthesis

## Abstract

Among eukaryotes, alternative splicing (AS) plays a role in mechanisms involved in processes such as regulation, development, and stress response. In animals, AS mainly functions in tissue development, whereas in plant species, AS plays a major role in stress response, a function additionally mirrored in microalgae. The latter species are highly valued for their ability to produce a variety of useful compounds. Furthermore, their productivity is directly intertwined with stress response, placing the mechanisms behind it in the spotlight. As stress can spur an increased production of pigments, lipids, fatty acids, and carbohydrates utilized in the synthesis of products such as nutraceuticals, pharmaceuticals, and biofuels. Delving into microalgae, we assess AS processes and the regulation of various developmental stages and stress conditions. Additionally, cyanobacteria also have high economic value. As prokaryotes with the ability to undergo self-splicing, research focus has promoted the phylum’s use in biotechnology to catalyze protein splicing. Although self-splicing and AS are two different types of splicing processes, there are some connections between them. For instance, the small nuclear RNA required for AS originates from group II introns. Therefore, this review focuses on elaborating on two distinct but related topics: the AS of microalgae and the three main forms of self-splicing intervening sequences (group I introns, group II introns, and inteins) in cyanobacteria.

**Supplementary Information:**

The online version contains supplementary material available at 10.1007/s44307-025-00087-3.

## Introduction

Alternative splicing (AS) is a process that removes different non-coding regions of mRNA (introns) and fuses coding sequences (exons) together to create transcripts with varying combinations. AS can directly edit pre-mRNA leading to changes in protein expression (Liu et al. 2024; Wright et al. 2022). Thus, the pre-mRNA existing within a cell can eventually be translated and processed into different protein structures, diversifying the genome and proteome (Panahi & Hejazi 2020).

AS events are commonly placed into five categories: exon skipping (ES) or cassette exons, mutually exclusive exons (MXEs), intron retention (IR), alternative 5’- (Alt 5’), and 3’-terminal exons (Alt 3’) (Breitbart et al. 1987; Cartegni et al. 2002; Luo et al. 2019). However, some studies have reported only four types, with the exclusion of MXEs (Galante et al. 2004; McGuire et al. 2008). The first category, ES, involves skipping an exon located between two exons in mature mRNA (Cui et al. 2017). Meanwhile, MXE produces similarly shaped protein isoforms with slightly different transcripts by keeping one exon or group of exons and splicing out another (Lam et al. 2021). The next AS event, IR, involves the retention of one or multiple introns in mature mRNA (Monteuuis et al. 2019). Lastly, Alt 5’ and Alt 3’ feature an exon, located in its respective terminal, surrounded by a constitutive splice site and multiple alternative splice sites (Koren et al. 2007). The processed sequence serves as an extension that is either excluded or included in the mature mRNA strand (Koren et al. 2007). These categories of AS are observed at varying frequencies for different species.

Among eukaryotes, AS is involved in diverse functions. However, the overarching goal of AS, to produce a variety of transcripts from the genome and increase transcriptome diversity, remains. For eukaryotes, AS plays a role in gene expression, thus has broad functions within processes such as stress and DNA damage response (DDR) mechanisms (Syed et al. 2012﻿; Nimeth et al. 2020; Panahi & Hejazi 2020). In animals, AS events are generally dependent on tissue type and contribute functionally to the diversification of cells and tissues (Martín et al. 2021). AS in plant species is mainly controlled by stress, thus it plays a role in stress response (Martín et al. 2021; Guo et al. 2023). The varying roles of AS in eukaryotes were previously associated with their motile status. Due to the ability of animals to move away from stressors, they instead require AS to contribute to the diversification of their cells for adaptation (Martín et al. 2021). Whereas plants must stay stationary, thus it is vital for them to adapt their stress response mechanisms by AS (Martín et al. 2021).

AS plays essential roles in many cellular processes (Marquez et al. 2012; Aghamirzaie et al. 2013; Huertas et al. 2019). These processes include mechanisms for stress response, tissue diversification and regulation, and genomic diversification and regulation (Thatcher et al. 2014; Calixto et al. 2018; Chen et al. 2018; Dong et al. 2018). Furthermore, the frequency of AS events in plants vary from species to species and in differing environmental conditions. For example, in *Arabidopsis*, approximately 22% of genes or 42% to 61% of genes containing introns undergo AS (Wang & Brendel 2006; Filichkin et al. 2010; Marquez et al. 2012; Chamala et al. 2015). These numbers also differ depending on environmental factors, where the occurrence of AS can increase to impact about 83.4% of genes containing introns after abscisic acid treatment, a compound resembling a signal released as a response to stressors (Zhu et al. 2017). Thus, this variety emphasizes the importance of AS in the response mechanisms of different plants. Currently, blooming progress has been made in identifying AS types and events in land plants and microalgae (Bao et al. 2013; Gupta et al. 2015).

Photosynthetic bacteria, such as cyanobacteria, mainly undergo self-splicing with group I introns, group II introns, and inteins (Meng et al. 2007). Furthermore, these sequences can be modified and applied in biotechnology (Stevens et al. 2016; Caspi et al. 2003; Dassa et al. 2007). Group I introns and group II introns are present in both prokaryotes, and the mitochondria and chloroplasts of eukaryotes (Haugen et al. 2005). Among them, group II introns are regarded as the ancestors of the eukaryotic spliceosome’s components (Novikova & Belfort 2017). The recognition and invasion of mRNA by a new group II intron of *Lactococcus lactis* and the generation of chimeras through trans-splicing has enabled the bacteria to develop a new splicing pathway, similar to the spliceosome of eukaryotes (Johnston et al. 2018). Thus, further demonstrates the evolutionary connection between group II introns, spliceosomal introns, and the spliceosome.

Therefore, this review will profile relevant reports on splicing in photosynthetic algae, including the basic mechanism and differences in splicing systems between eukaryotes, the functional analysis of splicing during development, and the involvement of splicing in response to biotic and abiotic stresses, and the self-splicing of cyanobacteria.

## The importance of alternative splicing in eukaryotes

**Fig. 1:**
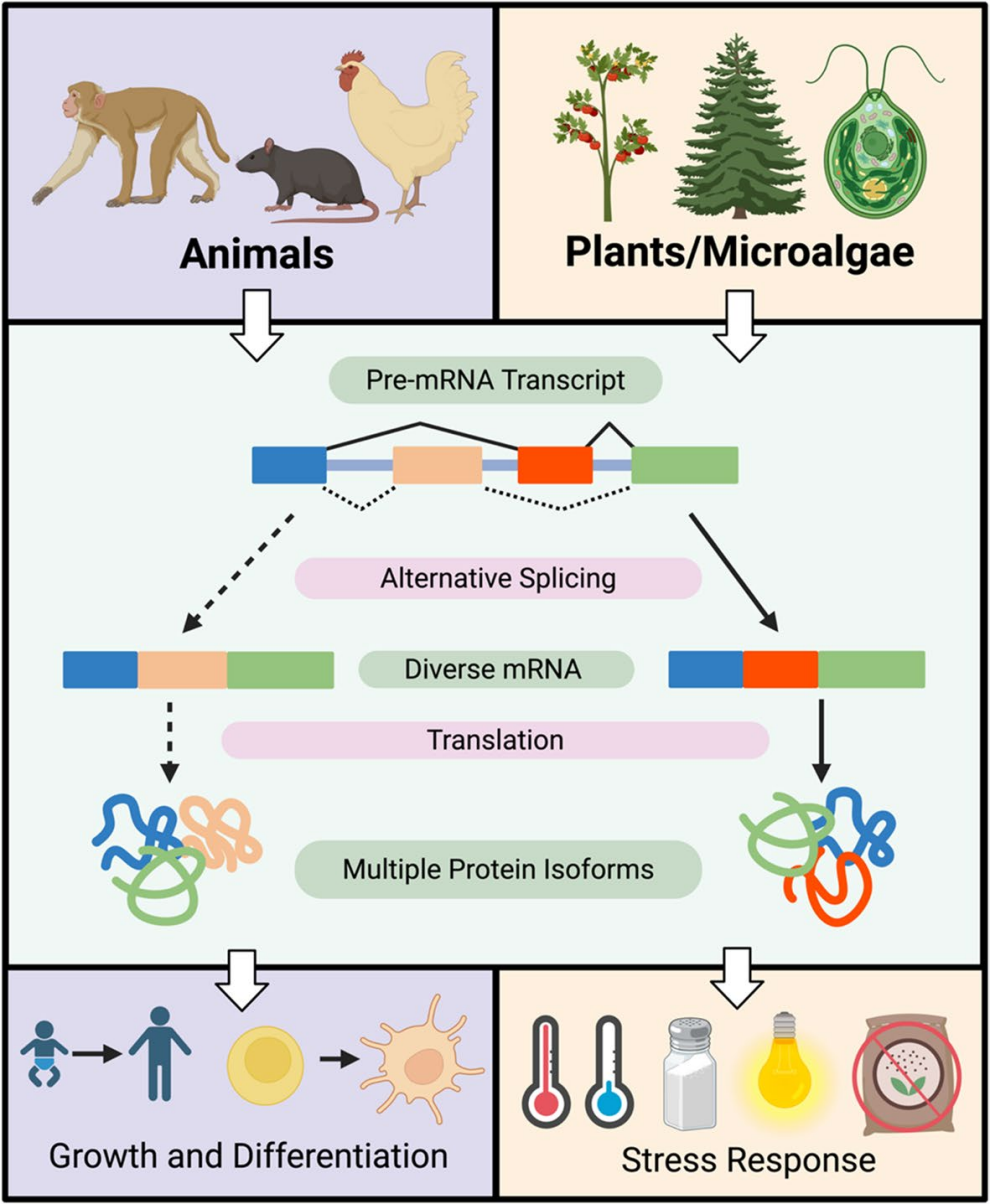
The comparison of animals, plants, and microalgae’s general use of alternative splicing (AS). Plants and microalgae, grouped with the former, tend to utilize AS as a response to stress, including conditions like extreme temperatures, salinity, light intensity, and nutrient deficiencies. Conversely, animals employ AS for growth and differentiation. A difference generally attributed to their motile status and thus the varying challenges they face to survive

Depending on eukaryotic species, the frequency of AS events can vary. Despite the similarities in function of AS events on gene structure, such as IR and ES, this diversity is observed when considering a variety of factors, including the life stage of the organism and the presence of environmental stressors (Grau-Bové et al. 2018). Furthermore, analyzing common AS events in eukaryotes reveals differences in the function of AS for animals, plants, and microalgae (Fig. 1).

In animals, the most common AS event type to occur tends to be ES (Grau-Bové et al. 2018). Further along the evolutionary timeline, bilaterian organisms’ reliance on ES increases drastically, where current animal species exhibit the highest frequency of ES, compared to the predicted frequency in extinct ancestral species (Grau-Bové et al. 2018).

The high frequency of ES is attributed to animals’ growth, differentiation, and movement (Grau-Bové et al. 2018). Wherein the event AS does not function as intended, cancer risk increases (Grau-Bové et al. 2018). Furthermore, the role of AS in organ development was validated by observing AS events in developing organs of a human, rhesus monkey, mouse, rat, rabbit, opossum, and chicken (Mazin et al. 2021). Firstly, revealing developmentally dynamic alternative splicing (devAS), or events with large changes in percentage spliced-in (PSI) value, were more highly preserved through evolution compared to nondynamic AS events (Mazin et al. 2021). Additionally, factors such as development stage and organ type can impact the frequency of AS events (Mazin et al. 2021).

In animals, the occurrence of some AS events are influenced by their environment. Where the process plays a role in adaptive plasticity in response to seasonal changes in *Bicyclus anynana*, which may also be reflected in wild butterfly populations (Steward et al. 2022). Furthermore, the regulation of AS has been attributed to the diversification of animal species like fish (Jacobs & Elmer 2021). For instance, in Arctic charr, AS is credited for the rapid evolution of several populations in separate localities (Jacobs & Elmer 2021). Additionally, the response to stress environments like high altitude is dictated by AS events. As high-altitude stress on Tibetan sheep negatively impacts fertility, and the increased of IR and decreased ES frequencies were responsible for fertility changes in the stressed sheep (Li et al. 2022). Furthermore, 917 differential AS events were caused by high-altitude stress alone (Li et al. 2022).

In comparison to animals, plants usually rely on IR (Grau-Bové et al. 2018). A characteristic of the group contributed to their relatively distinct survival requirements (Martín et al. 2021). As IR in plants, had been linked with growth, development, and stress response (Grau-Bové et al. 2018).

Similar to animals, plants can utilize AS for stress response. For *Arabidopsis thaliana*, under stress with either abiotic or biotic origins, AS is responsible for regulating translation as a response (Martín et al. 2021). A mechanism also mirrored in lilies (Wu et al. 2019).

In some plant species, heat stress transcription factors (HSFs) play a regulatory role in heat stress response (Li et al. 2014, 2024; Wu et al. 2019). Lily breeds, after encountering heat stress, will accumulate an alternatively spliced variant of *LlHSFA3B*, a thermal resistance gene, named *LlHSFA3B-III* (Wu et al. 2019). Furthermore, the resulting protein, LIHSFA3B-III, influences the activation of *LlHSFA3A-I*’s resulting protein, LlHSFA3A-I, a compound involved in the organism’s sensitivities to salinity and temperature (Wu et al. 2019). Therefore, by increasing the presence of *LlHSFA3B-III*, lily plants can increase their high temperature and salinity tolerance through control of LlHSFA3A-I (Wu et al. 2019). Thus, is one example of AS’s functionality in a largely ornamental species; however, the importance of AS is also shared among other largely cultivated species.

Many agriculturally impactful crops utilize AS in stress adaptation. For example, in wheat, AS regulates salinity stress response (Guo et al. 2020). Wherein AS events significantly edited 11,141 genes in reaction to salt stress (Guo et al. 2020). Additionally, in tea plants, AS processed terpene synthase (*CsTPS1-AS*) is mainly activated as a defense against pathogen infection (Jiang et al. 2023; Xie et al. 2023a, b). Whereas drought stress response’s efficacy relies on the production of isoforms synthesized by AS events of the circadian clock-associated 1 gene in maize and vegetative growth to reproductive growth transition factor gene in *Arabidopsis* (Tian et al. 2019; Chen et al. 2024). Thus, to respond to stress factors, many plant species rely on AS.

Beyond stress response, AS can contribute to metabolite synthesis and tissue development in plants. In *Camellia sinensis*, widely known as the tea plant, AS plays a role in the development and regulation of flavonoid synthesis, a molecule credited for the taste of tea (Zhu et al. 2018). For a functioning flavonoid processing pathway, AS is required to process specific structural genes, like leucoanthocyanidin and anthocyanidin reductase, and MYB transcription factors (Zhu et al. 2018). Furthermore, within *C. sinensis*, some tissue-specific development mediated by AS occurred (Zhu et al. 2018). Where a set of AS genes were simultaneously expressed in tissues with similar molecular characteristics (Zhu et al. 2018). Additionally, the inverse occurred, where large differences were observed in AS genes in tissues with distinct molecular makeups (Zhu et al. 2018). These results further indicate AS plays a variety of roles in plants.

The general purpose of AS in plant species is also mirrored in microalgae species. *Nannochloropsis oceanica* serves as a popular model organism for microalgae studies. However, research relating to long non-coding RNAs (lncRNAs), observed as moderators of AS in plants, has not been done extensively in microalgae (Wei et al. 2022; Zhao et al. 2024). lncRNAs usually play a role in growth, development, and stress response in plants (Wei et al. 2022). Therefore, *N. oceanica* lncRNA’s function under carbon dioxide fluctuation may elucidate its and AS’ function as a response mechanism (Wei et al. 2022). In total, 2051 AS events were identified to respond to CO_2_ treatment (Wei et al. 2022). However, these events were not linked with lncRNA, rather serving as evidence for AS’s crucial role in response to CO_2_ flux in *N. oceanica* (Wei et al. 2022).

The role of AS in eukaryotes varies depending on several factors, such as species, life stage, and environment. For instance, organisms within the animal kingdom can undergo AS for unique motives compared to plants. Furthermore, different species rely on a varied group of AS events. Where, dependent on the life stage of an organism, they require different AS mechanisms to diversify their transcriptome and proteome. Environmental conditions, such as seasonal changes or the presence of stressors, can also increase the frequency of AS on intron-containing genes as a method to respond and adapt to stress. Overall, AS remains an important mechanism for many eukaryotic species.

## Splicing regulation in microalgae

For eukaryotes, AS increases proteomic diversity through transcriptome assembly. The purpose of AS remains the same in some microalgae, a group comprised mainly of photosynthetic unicellular eukaryotes. Like higher plants, microalgae have desirable characteristics. For instance, they can be cultivated on a large scale in a relatively short growth period. Furthermore, these organisms can produce a broad range of significant products, which has emphasized the importance of understanding their growth and production mechanisms. These valuable compounds include, but are not limited to, minerals, vitamins, protein, lipids and carbohydrates (Khemiri et al. 2022; Zdziebłowska et al. 2024; Qian et al. 2021; Yang et al. 2024). Microalgae lipids have potential in biofuel production (Raheem et al. 2015; Culaba et al. 2020; Karkala et al. 2021). Whereas, many of these products hold potential or have been investigated for the synthesis of nutraceuticals (Karkala et al. 2021; Khemiri et al. 2022; Zdziebłowska et al. 2024). Wherein microalgae, the synthesis of fatty acids, including omega-3 fatty acids such as eicosapentaenoic acid (EPA) and docosahexaenoic acid (DHA) can be beneficial for supplement production (Togarcheti & Padamati 2021). Also, microalgae can be utilized in the production of pharmaceuticals (Ahmed et al. 2014). Through employing their ability to synthesize pigments, such as astaxanthin and β-carotene (Ahmed et al. 2014). More importantly, the synthesis of these products can be improved under stressful growth environments (Mutschlechner et al. 2022; Qiao et al. 2021).

**Fig. 2:**
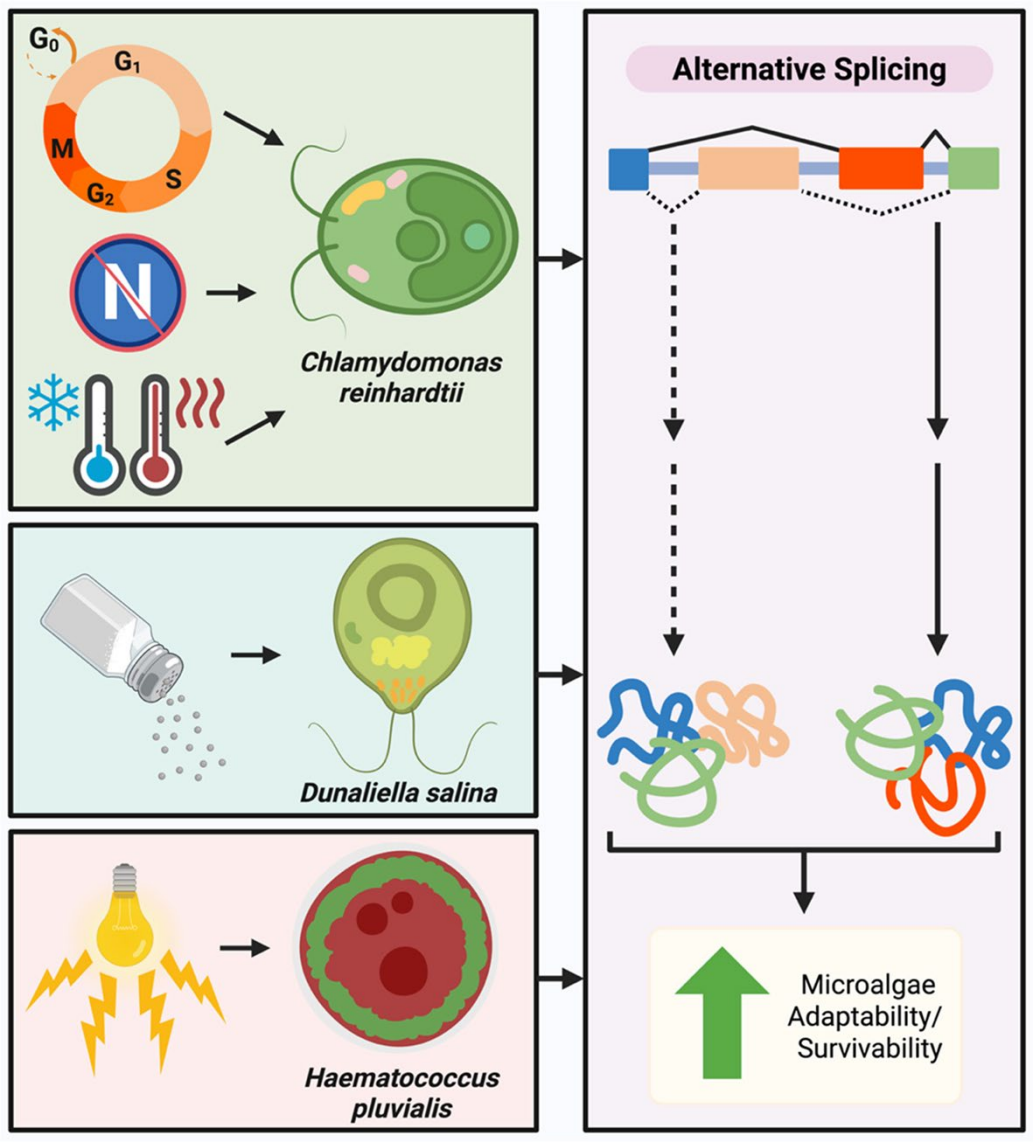
The usage of AS in microalgae stress response and cell cycle progression for three species. In *Chlamydomonas reinhardtii*, AS plays a role in cell cycle progression and several stress responses, such as extreme temperatures and nitrogen deficiency. In comparison, *Dunaliella salina* and *Haematococcus pluvialis* under salinity and light intensity stress, respectively, underwent AS

To take advantage of their productivity under stress conditions and relative ease to scale their culture, bioreactors, usually photobioreactors (PBRs), have been considered for large scale cultivation of microalgae (Do & Du 2024). These vessels allow users to customize diverse growth conditions to tailor the microalgae’s cultivation environment (Do & Du 2024). Therefore, revealing the role AS plays in their stress mechanisms may lead to greater understanding of these processes and influence how these organisms are employed in high scale production. However, the mechanisms behind stress response in microalgae have not been fully elucidated yet. The use and impact of AS events in several microalgae species and the current progress of microalgae AS research will be discussed further, with an emphasis on stress response (Table 1; Fig. 2).

**Table 1:** Role of alternative splicing in four model microalgae species

Species Name	Detection/Analysis Method	Role of Alternative Splicing	References
*Chlamydomonas reinhardtii*	RNA sequencing	AS involvement in cell cycle phases and gene expression	(Pandey et al. 2020)
	BLAT and Sircah	Involved in the regulation of gene expression, functional transcript rates, and protein function	(Labadorf et al. 2010)
	PASA, GMAP mapping, and BLAT	Increase transcriptome and proteome diversity, intron retention may function in gene expression regulation	(Raj-Kumar et al. 2017)
	DNA gel blot, sequencing	Production of CGE1 isomers, CGE1a is mostly present in standard growth conditions, and CGE1b is abundant after heat shock	(Schroda et al. 2001)
	RT-PCR, sequencing	Production of alternative transcripts of *THI4* and *THIC* for the regulation of thiamine synthesis	(Croft et al. 2007)
		Involved in proteome diversity for low temperature response	(Valledor et al. 2013)
	rMATs	Regulation of spliceosome and transporter pathways as a response to nitrogen deprivation	(Yang et al. 2022b)
*Dunaliella salina*		Production of variants of DsMEK1 that mediate glycerol production and decrease oxidative damage in response to salinity stress	(Tang et al. 2020)
	RT-PCR, Vector NTI 11.0 and BLASTx	Contributes to transcriptome and proteome diversity through the production of complex I 19-kD subunit isoforms in response to stress conditions	(Cao et al. 2010)
*Haematococcus pluvialis*	ASTALAVISTA	Intron retention is the main mode of alternative splicing	(Luo et al. 2019)
	PCR and MGAlign	In response to stress compounds, β-carotene ketolase gene 2 (*bkt2*) underwent AS in the untranslated region with no impact on the resulting amino acid sequence	(Lu et al. 2010)
*Nannochloropsis oceanica*	RNA-sequencing and Tophat, SUPPA, rMATs	AS events play a role in carbon dioxide fluctuation response	(Wei et al. 2022)

### *Chlamydomonas reinhardtii*

*Chlamydomonas reinhardtii* has an intron-rich genome with approximately 8.5 introns per gene, with roughly only 8% of genes being intron-less (Merchant et al. 2007; Labadorf et al. 2010; Weiner et al. 2018). In *C. reinhardtii* genes, approximately 50–55% exhibit alternative site selection (ASS), which includes events such as Alt 5’, Alt 3’, and ES (Labadorf et al. 2010; Raj-Kumar et al. 2017; Pandey et al. 2020). Whereas IR, the most prevalent AS type in the species, makes up approximately 29–50% of total AS events (Labadorf et al. 2010; Raj-Kumar et al. 2017; Li et al. 2018). Within the species’ transcriptome, frame-preserving and frame-disrupting splicing occur similarly frequently, with 1412 and 1555 events, respectively (Pandey et al. 2020). Frame-disrupting splicing exhibits a lower PSI value, which may suggest two possibilities: 1) The occurrence of nonsense-mediated decay (NMD), a process involved in removing mRNA with premature translation codons (PTCs); 2) In comparison to frame-preserving splicing, frame-disrupting splicing is rarer (Lin et al. 2018; Pandey et al. 2020). NMD may also contribute to the regulation of transcription in *Chlamydomonas* (Pandey et al. 2020). A result achieved by the introduction of an in-frame PTC with AS, inducing NMD in the transcript (Pandey et al. 2020). These results note the importance of AS in regulatory mechanisms in *Chlamydomonas* species.

In microalgae, AS events can occur at any stage of cell development. Within *C. reinhardtii*, fluctuating splicing patterns in AS events occurred through each phase of the cell cycle (Pandey et al. 2020). Of the splicing events to occur through *C. reinhardtii*’s cell cycle, approximately 29% of AS events overlap in light G1, S-M, and dark D1 phase (Pandey et al. 2020). The S-M phase occurs when the culture transitions from light to dark and continues into the dark phase (Pandey et al. 2020). However, some AS events were unique to a cell cycle phase (Pandey et al. 2020). For instance, within only the S-M phase, approximately 19% of AS events occurred (Pandey et al. 2020). Whereas about 17% of AS events were involved uniquely with the dark G1 phase, and nearly 10% of AS events were exclusice to the light G1 phase (Pandey et al. 2020). Furthermore, most genes in the *C. reinhardtii* genome did not display a significant change in gene expression as they progressed through the cell cycle phases, which indicates AS events are regulated separately (Pandey et al. 2020). Splicing events such as IR can illustrate this independent regulation. IR in *Chlamydomonas* can introduce PTCs, which can disrupt the RNA recognition motif (RRM) domain in mRNA, influence RNA recognition, and thus impact gene expression itself (Maris et al. 2005; Pandey et al. 2020). Since AS events occur throughout the cell cycle of *C. reinhardtii*, some of which are unique to certain stages, we can attribute one function of AS to cell development.

The mechanism of AS was also recognized to play a role in cold stress response. The cold environment instigated the expression of three splicing factors, splicing factor 3 subunit 1 and two splicing factors from the U1 snRNP family, which are involved in spliceosome activity (Valledor et al. 2013). Additionally, the low temperature treatment resulted in the eventual loss of splicing factors, such as the SC35-like factor and H/ACA snoRNP, and a decrease in expression of splicing factors, like the serine/arginine-rich mRNA (Valledor et al. 2013). These changes point towards the reconstruction of the spliceosome machinery to adapt to cold stress (Valledor et al. 2013). Thus, suggests AS is involved in producing protein isoforms to improve adaptivity for *C. reinhardtii* (Valledor et al. 2013). AS events also occur before structural ribosomal changes in the cytoplasm, mitochondria, and plastid, which result in the accumulation of the large and small subunits of the ribosome (Valledor et al. 2013). Thus, low temperatures result in the modification of *C. reinhardtii*’s proteome makeup through changes induced in translation and possible post-translational processes (Valledor et al. 2013).

In *C. reinhardtii*, one method to achieve gene expression enhancement is in transcribed open reading frames (ORFs) with intron-mediated enhancement (IME) (Baier et al. 2020). A process occurring without the influence of promoters or enhancers (Baier et al. 2020). RuBisCo small subunit 2 (rbcS2i1) has been previously identified to contain a robust IME-inducing intron for *C. reinhardtii* nuclear transgene expression, increasing expression by 5.5-fold (Baier et al. 2020). The IME capabilities of 13 exogenous introns, with varying functionalities, in *C. reinhardtii* were tested through insertion into the organism, revealing 11 of these sequences reduced expression levels (Baier et al. 2020). However, the result was attributed to the inclusion of foreign transgenes, sequences with no intron boundaries present in native or endogenous introns (Baier et al. 2020). Thus preventing recognition and, possibly, IME induction (Baier et al. 2020). The introduction of exogenous introns containing splice sites similar to native splice sites found in *C. reinhardtii*, resulted in high IME levels (Baier et al. 2018, 2020). Thus, the introduction of transgenes can impact expression. Analysis with a broader perspective reveals the processing of mRNA plays a role in controlling gene expression, thus also emphasizing the importance of AS in *C. reinhardtii*.

The transcription factor IIS (TFIIS) is related to the elongation function of RNA polymerase II and contributes to stress response and adaptation in plants (Szádeczky-Kardoss et al. 2022). Furthermore, the function of TFIIS is conserved in plants, and a gene coding for a TFIIS-like protein in *C. reinhardtii* bears similarities to the *tfIIs* gene found in *A. thaliana* (Szádeczky-Kardoss et al. 2022). Although the mechanism of TFIIS has not been further elucidated in *C. reinhardtii*, studies on *Arabidopsis* have revealed the importance of TFIIS function in heat stress and its impact on AS events (Szádeczky-Kardoss et al. 2022). As in *Arabidopsis* under heat stress, IR remained the most popular AS type (Szádeczky-Kardoss et al. 2022). Furthermore, without TFIIS, AS activity in genes was significantly affected (Szádeczky-Kardoss et al. 2022). Although these results were observed in *Arabidopsis*, due to the similarities of structure between the conserved TFIIS, the impact of TFIIS-like protein on AS may also be observed in *C. reinhardtii*, which may further point towards the regulatory influence of AS in heat stress response (Szádeczky-Kardoss et al. 2022).

In *C. reinhardtii*, stress response is also activated through nitrogen deprivation, a type of nutrient deficiency. Under these conditions, 3500 genes in the organism’s transcriptome underwent AS, totaling 5806 AS events (Yang et al. 2022b). These events were heavily involved in the regulation of spliceosome genes and transporter pathways, which play a role in mature mRNA assembly and the transportation of molecules, respectively (Yang et al. 2022b). Furthermore, in nitrogen starvation response, AS may be involved in decreasing the activity of the citrate cycle and fatty acid degradation pathways to contribute to the regulation of triacylglycerol (TAG) (Yang et al. 2022b). Additionally, AS genes were found in peroxisome-related regions of the cell, suggesting that AS is involved in the modification of membrane lipids (Yang et al. 2022b). Lastly, AS regulates protein degradation, as genes associated with AS and involved in amino acid and peptide metabolism were induced after nitrogen deprivation (Yang et al. 2022b). Generally, AS plays a role in a variety of nitrogen starvation responses in *C. reinhardtii*, including gene regulation, TAG accumulation, lipid structure, and amino acid and peptide metabolism (Yang et al. 2022b). Thus further provides evidence for the utilization of AS in diverse cellular functions for stress response mechanisms in *C. reinhardtii*.

### *Dunaliella salina*

*Dunaliella salina* is a unicellular green algae species, popular in industrial settings due to its halotolerant capabilities and high β-carotene productivity (Olmos-Soto et al. 2002; Wang et al. 2012). In comparison to *C. reinhardtii*, the *Dunaliella* species are alike morphologically, apart from the absence of a cell wall (Wang et al. 2012). Their similarities include the number of exons per gene, which remains roughly equivalent (Polle et al. 2020). However, the genome of *D. salina* (343.7 Mbp) is approximately three times larger than *C. reinhardtii* (111.1 Mbp) (Polle et al. 2020). This difference is mainly associated with intron presence, as the genome of *D. salina* contains 53% introns; however, in *C. reinhardtii*, 30% of the genome is composed of introns (Polle et al. 2020). The *D. salina* genome itself is composed of approximately 60% non-coding DNA (Smith et al. 2010). Besides genome size, contrasted to *C. reinhardtii*, the average gene length of *D. salina* is two-fold longer (Polle et al. 2020). Yet, on average, the exon length is shorter in *D. salina* (227 nt) than *C. reinhardtii* (366 nt) (Polle et al. 2020).

The high intron density of *D. salina* is also observed in its mitochondrial DNA (mtDNA), with about 1.5 introns per gene, and plastid DNA (ptDNA), with around 0.4 introns per gene (Smith et al. 2010). In the former, 58% is non-coding DNA, both intergenic and intronic DNA (Smith et al. 2010). Whereas in the latter, 65.5% of the genome consists of non-coding DNA (Smith et al. 2010). Of the percentage of non-coding DNA in mtDNA, 29.5% is intergenic and 28.5% is intronic (Smith et al. 2010). Whereas in the non-coding regions in ptDNA, 52% is intergenic and 13.5% is intronic (Smith et al. 2010). In mtDNA, 18 group I introns were identified, of which two sequences possess intronic ORFs (Smith et al. 2010). In ptDNA, 43 introns were identified (Smith et al. 2010). Of these introns, 36 were localized in genes and within this group, 17 introns had ORFs, 35 were group I introns, and one was identified as a group II intron (Smith et al. 2010). The seven other introns were group II introns found in intergenic regions, none of which contained ORFs (Smith et al. 2010). Of the 43 introns within ptDNA, 11 were inverted repeats or duplicates, and the remaining 32 were recognized as single-copy introns (Smith et al. 2010). Additionally, in intergenic ptDNA, eight nonfunctional pseudo-ORFs, or fragments of intronic ORFs, were identified (Smith et al. 2010). Their nonfunctional status was attributed to the presence of frameshift mutations within their sequence (Smith et al. 2010). However, the sequences of the eight pseudo-ORFs resemble groups I and II introns identified to code for RNA processing machinery such as the maturase and endonuclease (Smith et al. 2010).

*D. salina* can survive in diverse salinity concentrations; therefore, it is an ideal model for salt tolerance investigations (He et al. 2020; Polle et al. 2020). *D. salina* can quickly shrink or expand itself in response to salinity changes in the environment, making use of the cell wall-less nature of the species (Wang et al. 2012; Polle et al. 2020; Tang et al. 2020). In addition to a physical response to salinity, *D. salina* can employ other salt response strategies (He et al. 2020). One method includes an increase in spliceosome processing, a complex involved in post-transcriptional regulation (He et al. 2020). Thus, may allow the organism to manage genes critical for hypersaline stress response (He et al. 2020).

Like many algae species, *D. salina* can induce palmella formation, a vegetative state, under reduced salinity and hypersaline conditions (Wei et al. 2017; Novosel et al. 2022). Upon palmella formation, RNA processing-related proteins’ abundance was reduced, possibly contributing to a decrease in pre-mRNA splicing and localization (Wei et al. 2017). Whereas the G-patch domain-containing protein increased in abundance, a notable observation as the protein is involved in RNA recognition, binding, and splicing (Wei et al. 2017). Furthermore, the DEAD-box helicases (DBH), involved in RNA processing, were also altered in response to changes in salinity (Wei et al. 2017). Additionally, the phosphorylation of T72, a portion of RNA Polymerase Subunit 8 (RPB8), occurs (Wei et al. 2017). T27 is in the oligonucleotide/oligosaccharide-binding (OB)-fold nucleic acid binding domain, thus functions in nucleotide recognition (Wei et al. 2017). Although the purpose of phosphorylating RPB8 has yet to be uncovered, its increase would impact RNA polymerase III assembly and activity (Wei et al. 2017). RPB8 is also associated with the PRP1 splicing factor (PRP1), the phosphorylation of which declined after the palmella stage was achieved (Wei et al. 2017). Which may note a decrease in spliceosome activation, as PRP1 phosphorylation is involved in the process (Wei et al. 2017). Furthermore, suggesting a directly proportional relationship between decreased PRP1 phosphorylation, spliceosome activity, and, possibly, AS activity (Wei et al. 2017).

Under hypersaline conditions, additional survival strategies employed by *D. salina* include an increased expression of the MEK1 gene (Tang et al. 2020). *D. salina* MEK1 gene (DsMEK1) can undergo AS to produce two protein variants: truncated DsMEK1-X1 and full-length DsMEK1-X2, approximately 1000 bp and 1400 bp, respectively (Tang et al. 2020). The latter variant can work with DsMAPK1 and DsMAPKKK1/2/3/9/10/17 (Tang et al. 2020). Further analysis of the alternatively spliced DsMEK1 gene revealed, for DsMEK1-X1, four exons were spliced out, whereas, for DsMEK1-X2, 13 introns were removed (Tang et al. 2020). Under salt stress, DsMEK1-X2 is the preferred spliced variant; thus, it is over-expressed, resulting in an accumulation of glycerol in *D. salina* cells, which facilitates osmotic regulation, counteracting the hypersaline conditions (Ben-Amotz & Avron 1973; Tang et al. 2020). The truncated DsMEK1-X1 features a portion of the protein kinase and NTF2 domain, thus preventing strong protein interactions (Tang et al. 2020). Additionally, DsMEK1-X1 is the preferred spliced variant of DsMEK1 when *D. salina* undergoes salinity-induced oxidative stress (Tang et al. 2020). Thus, there is a reliance on AS isoforms in regulation and function diversity in the DsMEK1 gene originating from *D. salina* (Tang et al. 2020). Furthermore, four transcripts encoding the subunit 19KD of complex I were also isolated from *D. salina* (Cao et al. 2010). However, the functions of these four transcripts have not been reported yet.

### *Haematococcus pluvialis*

*Haematococcus pluvialis* is a unicellular freshwater green microalga renowned for its ability to synthesize high amounts of astaxanthin, an antioxidant carotenoid (Luo et al. 2019; Sun et al. 2022; Zhang et al. 2020). The potent antioxidant abilities of astaxanthin are attributed to the presence of a conjugated double bond, unsaturated keto, and hydroxyl groups present in its molecular structure (Sun et al. 2022). In comparison to naturally sourced β-carotene and vitamin E, astaxanthin is 10 and 550 times, respectively, more efficient as an antioxidant (Sun et al. 2022). As a result, astaxanthin accumulation serves as an anti-stress mechanism against free radical damage and oxidative damage (Lu et al. 2010; Ren et al. 2021a, 2021b; Zhang et al. 2020).

The current draft genome of *H. pluvialis* is 669 Mb in length, and contains 18,545 genes, with an average length of 8.7 kb per gene (Luo et al. 2019). Within the genome, 32.2% were transposable elements (Luo et al. 2019). Furthermore, transcriptome analysis of *H. pluvialis* revealed the total length of the entire transcript is approximately 96 Mb with a total of 157,035 mRNA transcripts, averaging 611 bp per transcript, and containing a GC content of 58.96% (Li et al. 2019). Within *H. pluvialis*, four types of AS can be observed: IR, ES, Alt 3’, and Alt 5’ (Luo et al. 2019). Of the four splicing mechanisms, IR occurred the most frequently (Luo et al. 2019).

As a reaction to undesirable environmental conditions, *H. pluvialis* accumulates fatty acids, one of the highly prized products of microalgae biomass (Zhang et al. 2020). The ∆12 fatty acid desaturase gene (*HpFAD2*) is involved in linoleic acid production, the first step of polyunsaturated fatty acid (PUFA) synthesis, thus functions in response mechanisms (Zhang et al. 2020; Ren et al. 2021b). *HpFAD2* is a point of interest to elucidate the production of PUFAs in *H. pluvialis* (Zhang et al. 2020). *HpFAD2* was observed to have an ORF, 1137 bp long, with the ability to code for 378 amino acids (Zhang et al. 2020). The entirety of *HpFAD2* contains nine introns scattered throughout the length of the gene (Zhang et al. 2020). The transcriptional levels of *HpFAD2* in *H. pluvialis* were observed to increase after the introduction of light stress, possibly implying higher rates of AS events (Zhang et al. 2020).

### The alternative splicing of other microalgae

The advancement of sequencing technology has contributed to elucidating the AS activity of other microalgae species. *Akashiwo sanguinea* can cause algal blooms and result in significant economic losses (Chen et al. 2022). Further analysis of the species with single-molecule real-time sequencing technology revealed 3137 AS events occurred within the full-length transcript (Chen et al. 2022). Furthermore, RNA-seq identified 2030 new loci within the genome of brown algae *Ectocarpus* (Cormier et al. 2017).

Additionally, improvements in sequencing have increased the exploration of AS in diverse microalgae species cultivated in various cultivation conditions. When *Auxenochlorella protothecoides* shifted from autotrophic to heterotrophic conditions, AS significantly increased (Panahi & Hejazi 2020). Through RNA-seq analysis, 705 differentially expressed genes (DEGs) and 660 differentially abundant sequences (DAS) were identified (Panahi & Hejazi 2020). In addition, many genes involved in carotenoid synthesis were downregulated under heterotrophic conditions, and the frequency of AS events within these genes also changed (Panahi & Hejazi 2020). Thus, suggesting the splicing patterns of carotenoid synthesis pathways serve as a response to changing growth conditions (Panahi & Hejazi 2020).

Channelrhodopsin (CCR) is generally involved in the regulation of green algae’s light mediated cellular functions through the synthesis of light-induced signals (Karpova et al. 2024). The CCR gene, however, differs between microalgae species regarding function and resulting protein expression. In *Haematococcus lacustris*, the CCR gene is processed with AS to synthesize the Hl98CCR2 protein, a sequence with no ability to interact with light (Karpova et al. 2024). Whereas, a short fragment of *Bracteacoccus aggregatus* CCR gene, synthesizes the functional Ba34CCR protein (Karpova et al. 2024). Although, Hl98CCR2 may require specific conditions to produce light-induced currents, thus requires further study regarding its functionality (Karpova et al. 2024). It is possible, AS plays a crucial role in the light regulated cellular functions of microalgae (Karpova et al. 2024). However, the role of AS in non-model microalgae can be further studied. Despite its significance in growth and stress response, AS pathways within other species are scarce. Therefore, AS remains to hold broad prospects and significance within the study of microalgae.

## The main self-splicing intervening sequences in cyanobacteria

Cyanobacteria, or green–blue algae, are photosynthetic prokaryotic organisms usually found in a variety of terrestrial and aquatic environments (Walter et al. 2017). The term green–blue algae was a result of some species’ ability to produce phycocyanin, a bluish-green phycobiliprotein recognized to assist with photosynthesis (Avci & Haznedaroglu 2022; Roy et al. 2024). Phycocyanin is highly valued for commercial uses, including the creation of pharmaceuticals and nutraceuticals (Avci & Haznedaroglu 2022). Furthermore, cyanobacteria can be engineered to become a source of prized compounds, like isobutanol, which can be utilized in the synthesis of biofuel (Xie et al. 2023a, b). In cyanobacteria, three primary forms of self-splicing intervening sequences have been reported: group I introns, group II introns, and inteins (Ferat & Michel 1993; Dassa et al. 2007; Haugen et al. 2007; Meng et al. 2007; Shah et al. 2012; Pfreundt & Hess 2015) (Table 2).

**Table 2:** Presence of self-splicing introns in model cyanobacteria species

Species Name	Gene	Type of Self-Splicing Intron	Function	References
*Nostoc punctiforme*	DnaE	Split intein	Associated with quick protein trans-splicing	(Shah et al. 2012)
*Nostoc punctiforme* PCC73102	Ribonucleotide reductase (*RIR*)	Group I intron	RNA splicing to create functional RIR protein	(Meng et al. 2007)
*Nostoc* sp. PCC7120	DnaE	Split inteins	Splicing efficiency decreases in high temperatures	(Dassa et al. 2007)
			Protein splicing is influenced by the addition of NaCl	
*Synechococcus* sp. C9	Large subunit (LSU) rRNA	Three group I introns		(Haugen et al. 2007)
*Synechococcus lividus* strain C1	Large subunit (LSU) rRNA	Two group I introns		(Haugen et al. 2007)
*Anabaena* PCC7120	Pre-tRNA^Leu^	Group I intron	Precursor to ribozyme that cleaves RNA	(Zaug et al. 1994)
	Pre-tRNA^Leu^	Group I intron	Use varying RNA and ion interactions to stabilize the catalytic site in the intron core	(Lupták & Doudna 2004)
*Calothrix* PCC7601		Group II intron		(Ferat & Michel 1993)
*Calothrix* PCC7101		Group II intron		(Ferat & Michel 1993)
*Oscillatoria limnetica*	DnaE	Split inteins	Protein splicing efficiency decreases in high temperatures	(Dassa et al. 2007)
*Synechocystis* sp. PCC6803	DnaE	Split inteins	In its split formation, functions in trans-splicing and trans-cleavage	(Nichols & Evans 2004)
*Trichodesmium erythraeum*	*Tery_4732*	Group II twintron	Spliced form is a functioning mRNA	(Pfreundt & Hess 2015)
*Thermosynechococcus vulcanus*	DnaE	Split inteins	Protein trans-splicing increases in a long-term salinity environment	(Dassa et al. 2007)

Group I introns, a class of catalytic RNA, can splice themselves (Cech 1990; Meng et al. 2007). This group of introns have conserved secondary and tertiary structures, the former of which are comprised of pairing (P) segments, P1 to P10 (Burke et al. 1987; Cech 1990; Hausner et al. 2014). The naming convention of these segments starts at the 5’ splice-site duplex of the catalytic center’s sequence, labeled as P1 (Burke et al. 1987; Zaug et al. 1994). The following helices are denoted similarly based on their location sequentially after P1, differing in the numerical value following “P” (Zaug et al. 1994). Although these P segments are characteristic of group I introns, some introns may not contain a P2 region; however, they do contain P1 and P3-P10 regions (Burke et al. 1987; Meng et al. 2007).

**Fig. 3:**
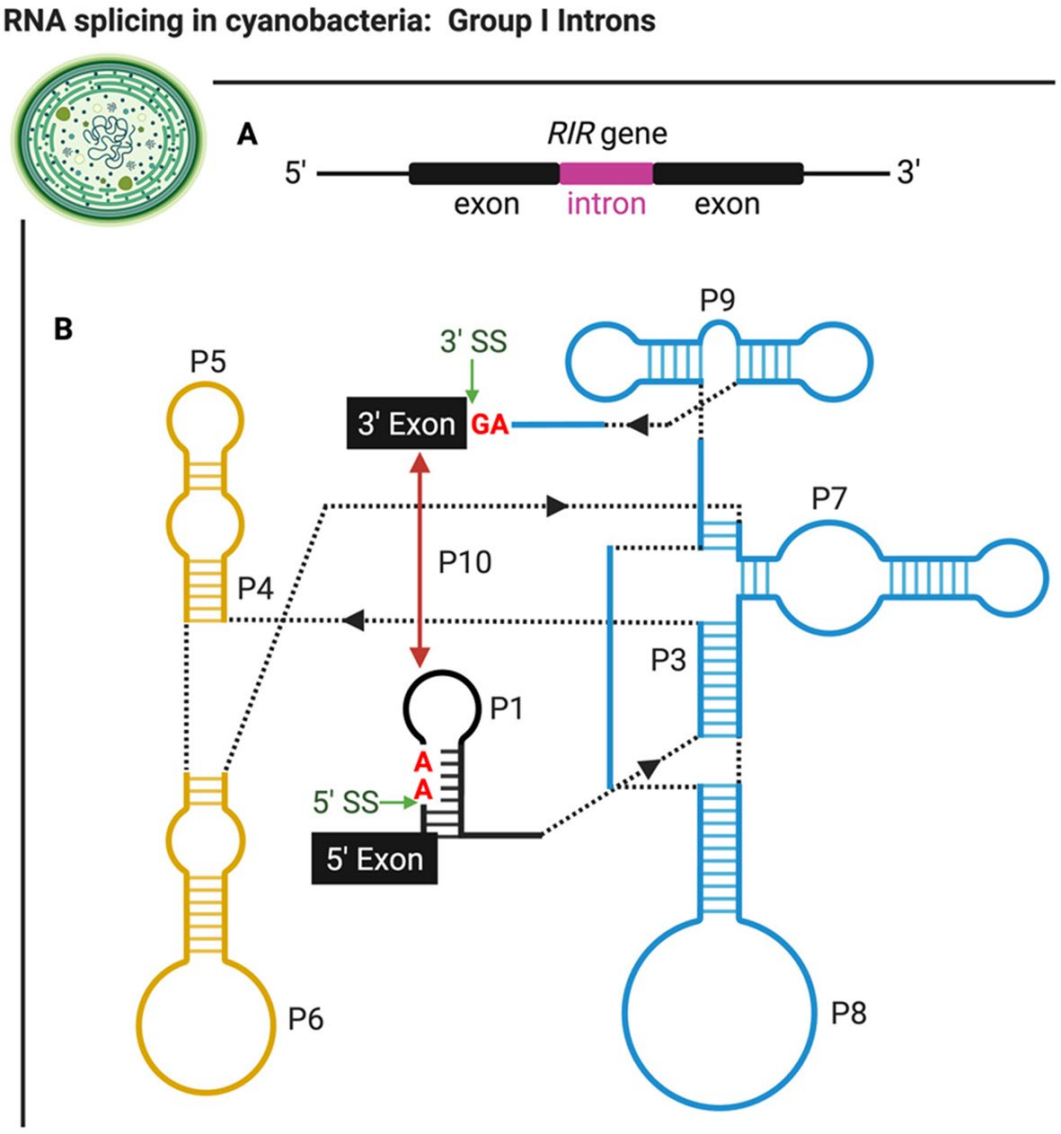
The ribonucleotide reductase (*RIR*) gene, an example of a gene containing a group I intron in cyanobacteria. **A**) The *RIR* gene **B**) The simplified folded structure of the *RIR* gene (Meng et al. 2007)

A group I intron was identified in the *Nostoc punctiforme* strain PCC73102 within the ribonucleotide reductase (*RIR*) gene (Fig. 3A), which is involved in RNA splicing to assemble a functional RIR protein (Meng et al. 2007) (Fig. 3B). The mechanism responsible for splicing is crucial in cyanobacteria, as the RIR protein is an enzyme involved in catalyzing the reduction of ribonucleotides to produce deoxyribonucleotides for DNA replication (Gleason & Olszewski 2002). Furthermore, self-splicing intervening sequences have been found within a variety of cyanobacterial *RIR* genes, including group I and II introns, and inteins (Meng et al. 2007). This further suggests the introns play a role in forming functional RIR protein structures (Meng et al. 2007).

Bacteria containing introns within ribosomal RNA (rRNA) typically possess only one, as opposed to green–blue algae, where multiple self-splicing rRNA group I introns have been reported (Haugen et al. 2007). Within cyanobacteria, several homing endonuclease genes (HEG)-containing group I introns in an rRNA sequence were identified (Haugen et al. 2007). The HEG serves to ensure the retention of itself, the intron within the genome, or both (Haugen et al. 2007). Thus, it contributes to the production of functioning proteins.

The cyanobacteria species *Anabaena* sp. strain PCC7120, contains a pre-tRNA^Leu^ with a group I intron (Zaug et al. 1994). Once this intron splices itself, it becomes a ribozyme, a catalyst for the cleavage of RNA (Zaug et al. 1994). The sequence was identified as a group I intron due to the presence of helical P segments that are characteristic of the group (Zaug et al. 1994). In comparison to the well-studied *Tetrahymena* (*Tet*) ribozyme with a 6 bp P1 sequence, the *Anabaena* ribozyme’s P1 is comprised of a 3-bp long internal guide sequence (IGS) (Zaug et al. 1994). Group I ribozymes which possess a longer IGS will experience a stronger interaction with the RNA substrate due to increased base-pairing (Zaug et al. 1994). As a result, under a single-turnover reaction with subsaturated RNA substrates, the *Anabaena* ribozyme mediates RNA cleavage at a slower rate compared to the *Tet* ribozyme (Zaug et al. 1994). Whereas under a multiple-turnover reaction with saturated RNA substrates, the *Anabaena* ribozyme allowed splicing to occur more readily than *Tet* ribozymes (Zaug et al. 1994). A shorter IGS implies less base pairing is occurring (Zaug et al. 1994). As a result, the substrate is held more loosely, which directly impacts its RNA splicing activity than ribozymes with longer P1 segments (Zaug et al. 1994).

The active site of group I introns is conserved and primarily composed of P3, P4, P6, and P7 stem-loop structures with the addition of their joining sequences (Lupták & Doudna 2004). These stem-loop structures form the binding site for the guanosine cofactor and helical splice site, serving as a part of the active site functioning in transesterification (Lupták & Doudna 2004). Despite high conservation in the active site, the individual subclasses of group I introns have developed unique mechanisms to stabilize their catalytic core (Lupták & Doudna 2004). Large introns use a peripheral domain to stabilize their core by forming a physical support for the active site (Lupták & Doudna 2004). In comparison, smaller introns stabilize the catalytic core with loop-helix interactions (Lupták & Doudna 2004). Therefore, despite being phylogenetically similar, different group I introns will employ unique tertiary interactions to stabilize the catalytic core for RNA transesterification in cyanobacteria (Lupták & Doudna 2004).

**Fig. 4:**
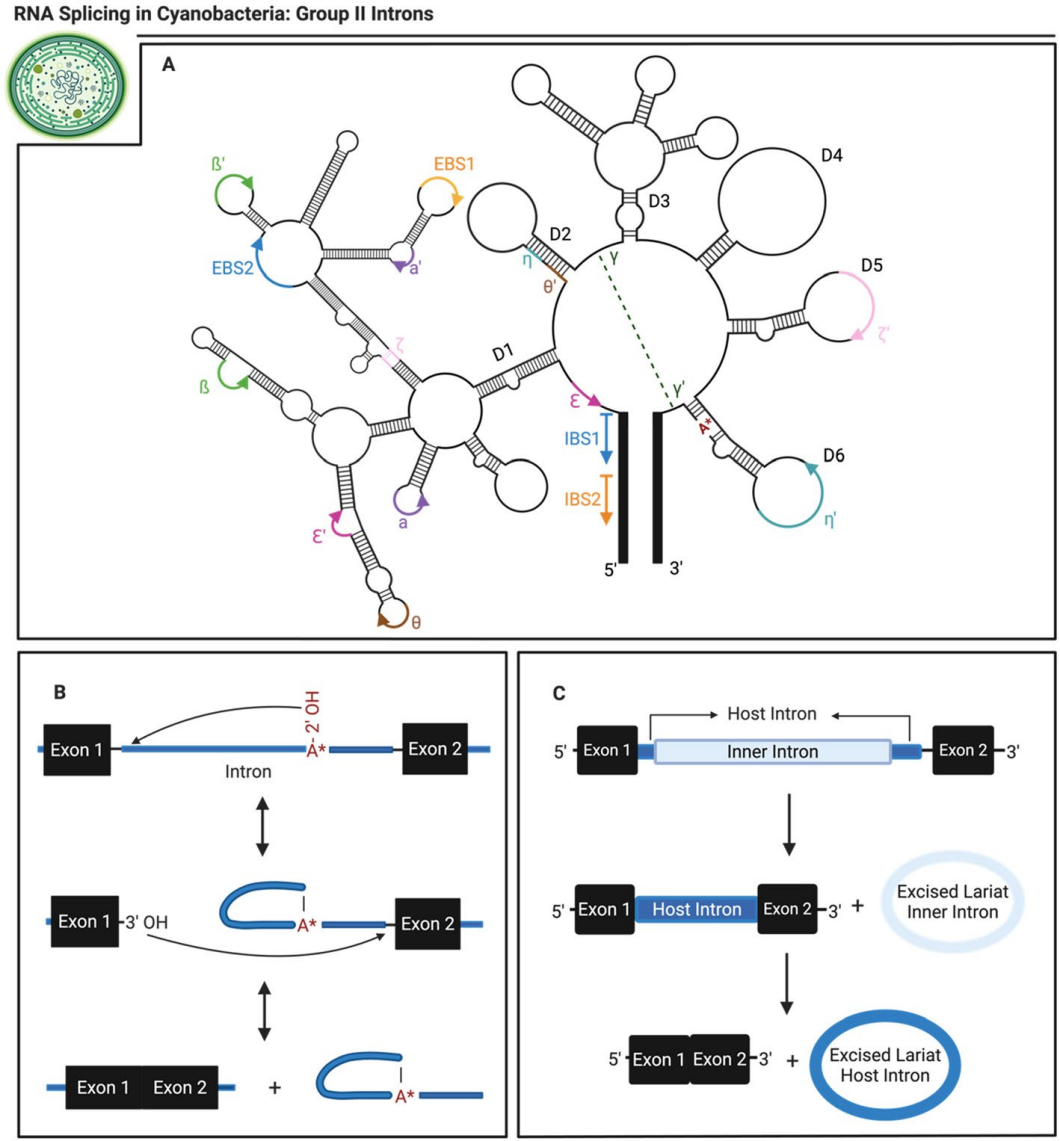
**A** Previously described structure of a group II intron ai5γ depicting its conserved domains (Jacquier & Michel 1987; Michel & Ferat 1995; Perlman & Podar 1996; Schmidt et al. 1998; Lehmann & Schmidt 2003; Novikova & Belfort 2017) **B**) Forward splicing mechanism of group II introns (Novikova & Belfort 2017; Haack et al. 2019) **C**) Proposed splicing mechanism of twintron found in cyanobacteria (Pfreundt & Hess 2015)

Group II introns have been found in a variety of organisms (Perlman & Podar 1996; Novikova & Belfort 2017) (Fig. 4A). Analogous to other introns, group II introns can be excised from the primary transcripts as lariats with a 2’-5’ phosphodiester bond (Ferat & Michel 1993) (Fig. 4B). Furthermore, they are believed to be evolutionary ancestors of eukaryotic spliceosomal components (Novikova & Belfort 2017). Structurally, these sequences usually contain an ORF (Pfreundt & Hess 2015). However, there have been cases where group II introns lack this region (Pfreundt & Hess 2015). In such situations, these introns are either spliced autocatalytically or utilize a splicing factor that is trans-encoded to aid in their removal (Pfreundt & Hess 2015).

Group II introns also include rarely observed introns within introns, also known as twintrons (Pfreundt & Hess 2015). In twintrons, the inner intron can be spliced independently of the larger host intron in which it resides (Pfreundt & Hess 2015) (Fig. 4C). For proper splicing and removal of the twintron, the inner intron must remove itself before the host intron can be properly spliced (Pfreundt & Hess 2015).

Comparable to group I and group II introns, inteins are a class of mobile, autocatalytic, self-splicing protein sequences (Dassa et al. 2007; Green et al. 2018). These sequences are considered intervening protein elements, as they are located between exteins, the protein equivalent of exons (Green et al. 2018). Inteins are capable of self-removal and ligation of flanking exteins, a process termed protein splicing, to produce a functional protein (Dassa et al. 2007; Green et al. 2018). There are two regions containing the splicing motif: the N-terminal splice site and the C-terminal splice site (Eryilmaz et al. 2014). For most contiguous inteins, splice sites are separated by a homing endonuclease or linker sequence (Eryilmaz et al. 2014). Thus, they tend to undergo *cis*-splicing (Eryilmaz et al. 2014). Alternatively, split inteins, or intervening protein elements, undergo *trans*-splicing (Dassa et al. 2007; Eryilmaz et al. 2014). As the sequence contains two separate sequences in their respective terminals, comprised of a C-intein with the C-extein, part of the eventual peptide, and a separate N-intein and N-extein (Dassa et al. 2007; Eryilmaz et al. 2014; Seo & Bang 2020). Thus, in split inteins, it is necessary to ligate the two exteins to synthesize a functioning protein (Seo & Bang 2020).

**Fig. 5:**
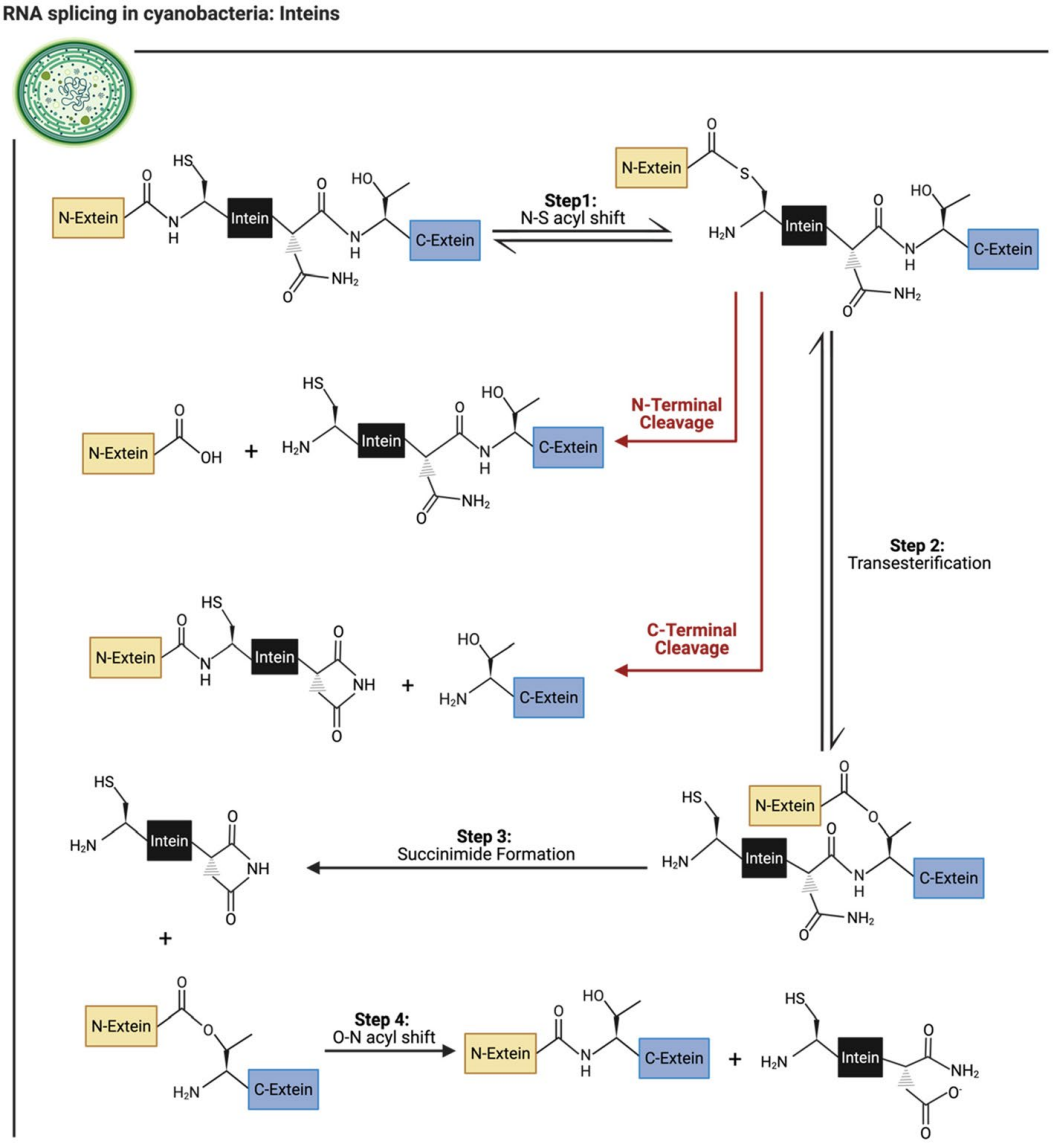
The proposed mechanism of intein self-removal described in several species, including bacteria and yeast (Chong et al. 1996; Shao et al. 1996; Xu & Perler 1996; Liu et al. 2014). The depicted intein is found in *Mycobacterium xenopi* (Liu et al. 2014). This peptide will undergo a four-step splicing sequence to result in the removal of the intein sequence and ligation of the two exteins. (Chong et al. 1996; Shao et al. 1996; Xu & Perler 1996; Paulus 2000; Liu et al. 2014; Mills et al. 2014; Ramsoomair et al. 2017)

Protein splicing is a four-step process (Chong et al. 1996; Shao et al. 1996; Xu & Perler 1996; Paulus 2000; Mills et al. 2014; Ramsoomair et al. 2017) (Fig. 5). The first step involves the conversion of the amide peptide bond into a thioester via a nucleophilic attack on the N-terminal splice junction amino acid’s thiol group (Chong et al. 1996; Shao et al. 1996; Xu & Perler 1996; Paulus 2000; Mills et al. 2014; Ramsoomair et al. 2017). Following this, the amino acid at the C-terminus splice junction will attack the newly formed thioester (Chong et al. 1996; Xu & Perler 1996; Paulus 2000; Ramsoomair et al. 2017). This step produces a branched thioester intermediate (Chong et al. 1996; Xu & Perler 1996; Paulus 2000; Mills et al. 2014; Ramsoomair et al. 2017). In this step, instead of thioester intermediates, oxygen esters can be formed, which is dependent on the amino acid present on the C-extein’s N-terminal (Ramsoomair et al. 2017). Continuing through the protein splicing mechanism, succinimide formation occurs, which includes cyclization of the intein’s C-terminal Asn (Chong et al. 1996; Xu & Perler 1996; Paulus 2000; Mills et al. 2014; Ramsoomair et al. 2017). Following this, the branched intermediate is cleaved, releasing the intein (Chong et al. 1996; Xu & Perler 1996; Paulus 2000; Mills et al. 2014; Ramsoomair et al. 2017). The newly spliced extein sequence then undergoes an O-N acyl shift, a spontaneous process that converts the ester bond, linking the two exteins, into an amide bond (Chong et al. 1996; Xu & Perler 1996; Paulus 2000; Mills et al. 2014; Ramsoomair et al. 2017). Whereas the aminosuccinimide on the C-terminus of the intein undergoes hydrolysis (Paulus 2000; Ramsoomair et al. 2017).

Within these splicing steps, regulation is crucial. Both the branched and linear thioester intermediates, can undergo N-terminal cleavage (Ramsoomair et al. 2017). This could occur by hydrolysis or thiolysis, and result in improper spicing (Ramsoomair et al. 2017). Additionally, if cyclization at the third step transpired prior to the formation of the branched ester, it could have resulted in improper splicing or C-terminal cleavage (Ramsoomair et al. 2017). As it can be inferred, the steps of splicing are tightly coordinated. As failure to regulate these steps would result in unproductive cleavage of either exteins’ terminals, thereby preventing the excision of inteins and splicing of the two exteins together (Ramsoomair et al. 2017).

Split inteins can serve as vital tools in biological engineering with their ability to quickly link two individual polypeptides (Stevens et al. 2017). In DnaE split inteins, the C-terminal region of the N-intein is a unique sequence in composition and length (Dassa et al. 2007). Split inteins are capable of self-associating due to their high affinity for one another (Dassa et al. 2007). Upon reassociating, the new complex can undergo protein splicing or the cleavage of its terminal intein (Dassa et al. 2007) (Fig. 5). Due to the presence of split inteins in cyanobacteria, they can be utilized to further study the activity of split inteins for applications in biotechnology and chemistry research through the synthesis of novel split inteins (Seo & Bang 2020).

Surrounding conditions can also play a role in splicing activity, as divalent metal copper ions can prevent trans-splicing of proteins through a double-lock reaction (Dassa et al. 2007). The copper ions would interact with either the N- or C-terminal cysteine residues of a split intein (Dassa et al. 2007). This interaction physically alters the intein’s active site by forming a protein-metal complex that prevents splicing and cleaving events (Dassa et al. 2007). Since divalent copper ions are a strong oxidizing agent, to restore splicing of the split inteins, they must first be chelated by metal chelators such as ethylenediaminetetraacetic acid (EDTA) (Dassa et al. 2007). Additionally, only chelating the copper ion would not restore normal function, as the N- or C-terminal cysteine would still be oxidized. Therefore, the protein from the previously formed complex must be reduced as well (Dassa et al. 2007). Other divalent metal ions such as zinc, cobalt, nickel, and magnesium can also potentially block splicing, though less effectively compared to divalent copper ions (Dassa et al. 2007).

Highly active split DnaE inteins in cyanobacteria can contain conserved amino acid regions (Shah et al. 2012). Divergence in these regions can be associated with low activity inteins (Shah et al. 2012). Though they are associated with facilitating high activity, these conserved amino acid residues reside outside the active site (Shah et al. 2012). Thus, the conservation of these amino acids may play a vital role in stabilizing the folded structure of the split intein (Shah et al. 2012). Therefore, they are associated with high levels of activity (Shah et al. 2012). Within cyanobacteria’s family of split DnaE inteins, the importance of conservation in a C-terminal amino acid lies in its ability to cyclize, which is a vital step in protein splicing as this removes the intein from the spliced extein structure (Ramsoomair et al. 2017). Additionally, the histidine residue at the penultimate intein position is usually conserved (Nichols & Evans 2004). The conservation of penultimate histidine is a result of its contribution to aspartic acid cyclization (Nichols & Evans 2004). However, as observed in *Synechocystis* sp. PCC6803, which contains a penultimate alanine, penultimate histidine can also be substituted into this position (Nichols & Evans 2004). Despite substitution, *Synechocystis* sp. PCC6803 displayed no increase in either frequency nor scope of splicing mechanisms (Nichols & Evans 2004). Thus, the combined effects of the conserved amino acid residues allow for meaningful and proper removal of inteins and splicing of exteins.

## Conclusions and perspectives

Functional in eukaryotes, AS is involved in a variety of regulatory processes. The main goal of AS is to increase transcriptome diversity, which increases proteome diversity. In turn, this process impacts areas like tissue development and stress response. For microalgae, AS has been credited largely with the latter.

The comparison between the AS events of microalgae with other eukaryotes revealed the AS activity of microalgae is more similar to those of plants. Among animals, ES usually is the most frequent AS event (Kim et al. 2007; McGuire et al. 2008; Martín et al. 2021), and the changes in AS events mainly manifest in their function on organ development (Mazin et al. 2021). While in plants and microalgae, IR is usually the most common AS event (Ner-Gaon et al. 2004; Wang & Brendel 2006; Wang et al. 2008; McGuire et al. 2008; Grützmann et al. 2014), and the changes in AS events are mainly attributed to the role of IR in growth and stress tolerance (Valledor et al. 2013; Zhu et al. 2017; Wu et al. 2019; Tang et al. 2020; Yang et al. 2022a, 2022b). Table 1 summarizes the methods for identifying AS events and the functions of AS in four model microalgae species.

With the development of sequencing technology, many methods have been employed to detect and analyze AS events. RNA-seq, including short-read RNA-seq (Illumina NGS) and long-read RNA-seq (PacBio and ONT), is a powerful tool for detecting AS (Ura et al. 2021, 2022). However, each method comes with disadvantages. Short-read RNA-seq can identify whether AS occurs inbetween two exons, but is unable to recognize AS within an entire transcript (Ura et al. 2022). Long-read RNA-seq can detect all AS events of the full-length transcript (Ura et al. 2022). However, it often amplifies non-specific regions of genomic DNA, thereby affecting the accuracy of AS detection (Ura et al. 2021, 2022). DNase I enzymes, with the ability to digest genomic DNA, or magnetic Oligo(dt) beads, capable of capturing the Poly(A) tail, can be introduced during sequencing to address genomic DNA (Ura et al. 2022). Thereby, improving RNA-seq's ability to identify AS events. Although third-generation sequencing provides longer reads, it is accompanied by high rates of error (Križanovic et al. 2018). PSI value represents the percentage of alternatively spliced exons’ homologous isoforms to the total expression levels of isoforms spliced both constitutively and alternatively (Kakaradov et al. 2012). Thus, it is a value used to analyze ES events. When ΔPSI is greater than 0.1, it is usually regarded as a strict criterion for confirming significant AS events (Lu et al. 2022; Qi et al. 2024). The methods for calculating PSI include MISO and rMATS, based on exons, and SUPPA2, based on isoforms (Kakaradov et al. 2012; Lin & Krainer 2019; Qi et al. 2022). However, none of these can be used for the detection of multiple exon skipping (Lin & Krainer 2019). Furthermore, tools such as IRFinder, to detect IR events, and Whippet, to analyze complex AS events, have been developed to address bioinformatic challenges associated with AS analysis (Middleton et al. 2017; Sterne-Weiler et al. 2018). The advancement of sequencing technology has solved some challenges related to identifying AS events in sequencing data. However, more comprehensive analysis methods are required.

Algae, such as microalgae and cyanobacteria, are valuable due to their rapid growth rate, tolerance to stress, and their productivity. Wherein advancements to bioreactor design have arisen with the rise of 3D printing. Due to their design flexibility, 3D-printed bioreactors can potentially cater to specific microalgae species’ growth requirements thus can improve productivity (Sun et al. 2024). Through employing new growth technologies for microalgae cultivation, it is possible to increase the utilization of microalgae productivity in a diverse range of industries (Zhu et al. 2024).

In addition to improving cultivation systems, stress can contribute to enhancing the accumulation of desirable products in microalgae (Zhang et al. 2024; Mutschlechner et al. 2022; Qiao et al. 2021). Thus, revealing the relationship between stress response and AS is of great importance as it may elucidate additional methods for the efficient employment of the organisms’ productivity. For instance, in the studies of *C. reinhardtii*'s response to low temperature and nitrogen deficiency, the role of AS was revealed, through influencing the expression of related genes (Valledor et al. 2013; Yang et al. 2022b). *D. salina* is a favored species due to its salt tolerance and pigment productivity (Olmos-Soto et al. 2002; Wang et al. 2012). Furthermore, the species utilizes AS to help tolerate high salt environments (He et al. 2020; Wei et al. 2017). Some mechanisms for its successful salt stress response include altering spliceosome processing, increasing the abundance of machinery involved in RNA recognition, and synthesizing spliced variants of relevant genes like DsMEK1-X2 (Ben-Amotz & Avron 1973; Wei et al. 2017; He et al. 2020; Tang et al. 2020). Lastly, *H. pluvialis* may have a higher rate of selective splicing events when subjected to light stress (Zhang et al. 2020). These findings shed light on the mechanisms utilized by microalgae in the presence of stressors, thus possibly elucidating the mechanisms behind morphological and productivity changes in stressed microalgae. Focusing on a handful of model species, AS’s regulatory role remains, functioning in formulating responses to temperature extremes, nutrient deficiencies, and salinity stress.

Although gene editing in algae is still in the early stages, some efficient protocols for transformation systems based on CRISPR-Cas9 have been established. Currently, genetically modifying algae to increase productivity efficiency has been explored. Industrial green algae *Coccomyxa* sp. strain KJ is an excellent candidate for biofuel feedstock (Yoshimitsu et al. 2018)﻿. As, in a nitrogen deficient environment, the strain can synthesize triacylglycerols comprising more than 60% of dry weight (Yoshimitsu et al. 2018). Furthermore, cultivation requirements are minimal, since the species only requires a small volume of mineral-based culture medium (Yoshimitsu et al. 2018). To synthesize *Coccomyxa* sp. strain KJ mutants with lipid productivity, recombinant CRISPR-Cas9 and guide RNA can be utilized to knockout the *KJFTSY* gene (Yoshimitsu et al. 2018). *Nannochloropsis gaditana* has high lipid productivity under nutrient deficient conditions, but the same conditions also lead to slow growth and reduced productivity overall (Ajjawi et al. 2017). CRISPR-Cas9 can edit transcription factors which negatively regulate lipid synthesis (Ajjawi et al. 2017). The resulting mutants produce twice as much lipids as the wild type without affecting growth (Ajjawi et al. 2017).

These findings shed light on the mechanisms utilized by microalgae in the presence of stressors, thus possibly elucidating the processes behind morphological and productivity changes in stressed microalgae. Furthermore, the self-splicing mechanisms in cyanobacteria have also been revealed in detail, including the role of group I and II introns, and inteins in the species. Therefore, exploring the modification of AS or self-splicing of microalgae and cyanobacteria, respectively, to target their stress tolerance utilized in tandem with bioreactors, may contribute to advancements in increasing productivity.

## Supplementary Information

Supplementary Material 1.

## Data Availability

All data generated or analyzed during this study are included in this published article.
