# Supplementary material for: Pre-mRNA splicing and its regulation in microalgae and cyanobacteria
Source: Adv Biotechnol (Singap). 2025 Dec 14;3(4):35. doi: 10.1007/s44307-025-00087-3 (PMC12702839; doi:10.1007/s44307-025-00087-3)
Supplement: Supplementary file 1 — Supplementary Material 1. [file 44307_2025_87_MOESM1_ESM.pdf]

SPRINGER NATURE

Change of authorship request form - Journals (post-acceptance)

Important information. Please read.

- This form should be used by authors to request any change in authorship (adding/deleting authors) including changes in corresponding authors. This form should not be used for name changes. Please fully complete all sections. Use black ink and block capitals and provide each author's full name with the given name first followed by the family name.

- By signing this declaration, all authors guarantee that the order of the authors are in accordance with their scientific contribution, if applicable as different conventions apply per discipline, and that only authors have been added who made a meaningful contribution to the work.

- Please note, in author collaborations where there is formal agreement for representing the collaboration, it is sufficient for the representative or legal guarantor (usually the corresponding author) to complete and sign the Authorship Change Form on behalf of all authors, **next to the added/removed author(s)**. **(Complete Section 3, followed by Section 6.)**
  In author collaborations where there is no formal agreement for representing the collaboration and **there are more than 10 authors**, one may sign for all, provided the signer appends correspondence that attests that each of the authors have agreed to the change **and the added/removed authors sign the form**. **(Complete Section 3, followed by Section 6.)**

- Please note, we cannot investigate or mediate any authorship disputes. If you are unable to obtain agreement from all authors (including those who you wish to be removed) you must refer the matter to your institution(s) for investigation. Please inform us if you need to do this.

- If you are not able to return a fully completed form within **5 days** of the date that it was sent to the author requesting the change, we may have to withdraw your manuscript. We cannot publish manuscripts where authorship has not been agreed by all authors (including those who have been removed).

- Incomplete forms will be rejected.

- Please return this form, fully completed, to Springer Nature. The Journal and/or Publisher will consider the information you have provided to decide whether to approve the proposed change in authorship. We may choose to contact your institution for more information or undertake a further investigation, if appropriate, before making a final decision.

SPRINGER NATURE Springer Nature is one of the world's leading global research, educational and professional publishers, created in May 2015 through the combination of Nature Publishing Group, Palgrave Macmillan, Macmillan Education and Springer Science+Business Media.

Change of authorship request form - Journals (post-acceptance)

SPRINGER NATURE

**Manuscript ID no.: ADBT-D-25-00054R1**
**DOI (if available): 10.1001/s44307-025-00087-3**

Section 1: Please provide the current title of manuscript

Title: Pre-mRNA splicing and its regulation in microalgae and cyanobacteria

Section 2: Please provide the previous authorship, in the order shown on the manuscript before the changes were introduced. Please indicate the corresponding author by adding (CA) behind the name.

	First name(s)	Family name	ORCID or SCOPUS id, if available
1st author	Sally	Do	
2nd author	Yue	Liu	
3rd author	Henry	Huynh	
4th author	Yinggao	Liu	
5th author	Jing-Jing	Huang	
6th author	Wujiao (CA)	Li	
7th author	Mo-Xian (CA)	Chen	
8th author	Zhi-Yan (CA)	Du	
9th author			
10th author			

Please use an additional sheet if there are more than 10 authors.

SPRINGER NATURE Springer Nature is one of the world's leading global research, educational and professional publishers, created in May 2015 through the combination of Nature Publishing Group, Palgrave Macmillan, Macmillan Education and Springer Science+Business Media.

SPRINGER NATURE

Change of authorship request form - Journals (post-acceptance)

Section 3: Please provide a justification for change. Please use this section to explain your reasons for changing the authorship of your manuscript, e.g. what necessitated the change in authorship? Please refer to the journal policy pages for more information about authorship. Please explain why omitted authors were not originally included and/or why authors were removed on the submitted manuscript and/or why the corresponding author needs to be changed.

Dr. Jing-Jing Huang has requested to be withdrawn from the author list for personal reasons.

Section 4: Proposed new authorship. Please provide your new authorship list in the order you would like it to appear on the manuscript. Please indicate the corresponding author by adding (CA) behind the name. If the Corresponding Author has changed, please indicate the reason under section 3.

	First name(s)	Family name (this name will appear in full on the final publication and will be searchable in various abstract and indexing databases)	Affiliated institute	E-mail address
1st author	Sally	Do	University of Hawai'i at Manoā	sall924@hawaii.edu
2nd author	Yue	Liu	Nanjing Forestry University	2019110084@sdau.edu.cn
3rd author	Henry	Huynh	University of Hawai'i at Manoā	henryhuy@hawaii.edu
4th author	Yinggao	Liu	Shandong Agricultural University	liuyg@sdau.edu.cn
5th author	Wujiao (CA)	Li	Shenzhen Children's Hospital	hnnd059@gmail.com
6th author	Mo-Xian (CA)	Chen	Guizhou University	cmx2009920734@gmail.com
7th author	Zhi-Yan (CA)	Du	University of Hawai'i at Manoā	duz@hawaii.edu
8th author				
9th author				
10th author				

Please use an additional sheet if there are more than 10 authors.

SPRINGER NATURE Springer Nature is one of the world's leading global research, educational and professional publishers, created in May 2015 through the combination of Nature Publishing Group, Palgrave Macmillan, Macmillan Education and Springer Science+Business Media.

SPRINGER NATURE Change of authorship request form - Journals (post-acceptance)

Section 5: Author contribution, Acknowledgement and Disclosures. Please use this section to provide a new disclosure statement and, if appropriate, acknowledge any contributors who have been removed as authors and ensure you state what contribution any new authors made (if applicable per the journal policy). Please ensure these are updated in your proofs - <u>after approval of the change(s)</u> - as our production department will not transfer the information in this form to your manuscript.

New acknowledgements: Not applicable.

New Disclosures (financial and non-financial interests, funding): Not applicable.

New Author Contributions statement (if applicable per the journal policy): Not applicable.

State 'Not applicable' if there are no new authors.

SPRINGER NATURE Springer Nature is one of the world's leading global research, educational and professional publishers, created in May 2015 through the combination of Nature Publishing Group, Palgrave Macmillan, Macmillan Education and Springer Science+Business Media.

SPRINGER NATURE Change of authorship request form - Journals (post-acceptance)

Section 6: Declaration of agreement. *All* authors, unchanged, new and removed *must* sign this declaration.
(NB: Please print the form, (docu)-sign and return a scanned copy. Please note that signatures that have been inserted as an image file are acceptable as long as it is handwritten. Typed names in the signature box are unacceptable.) * Please delete as appropriate. Delete all of the bold if you were on the original authorship list and are remaining as an author.

	First name	Family name		Signature	Date
1st author	Sally	Do	I agree to the proposed new authorship shown in section 4 and the proposed change in corresponding author	[illegible]	
2nd author	Yue	Liu	I agree to the proposed new authorship shown in section 4 and the proposed change in corresponding author	刘月	
3rd author	Henry	Huynh	I agree to the proposed new authorship shown in section 4 and the proposed change in corresponding author	HH	
4th authors	Yinggao	Liu	I agree to the proposed new authorship shown in section 4 and the proposed change in corresponding author	[illegible]	
5th author	Jing-Jing	Huang	I agree to the proposed new authorship shown in section 4 **removal*of my name to the authorship list** and the proposed change in corresponding author	[illegible]	
6th author	Wujiao	Li	I agree to the proposed new authorship shown in section 4 and the proposed change in corresponding author	Wujiao Li	
7th author	Mo-Xian	Chen	I agree to the proposed new authorship shown in section 4 and the proposed change in corresponding author	[illegible]	

SPRINGER NATURE Springer Nature is one of the world's leading global research, educational and professional publishers, created in May 2015 through the combination of Nature Publishing Group, Palgrave Macmillan, Macmillan Education and Springer Science+Business Media.

SPRINGER NATURE

Change of authorship request form - Journals (post-acceptance)

	First name	Family name		Signature	Date
8th author	Zhi-Yan	Du	I agree to the proposed new authorship shown in section 4 and the proposed change in corresponding author	zhiyandu	
9th author			I agree to the proposed new authorship shown in section 4 **/and the addition/removal*of my name to the authorship list** /and the proposed change in corresponding author		
10th author			I agree to the proposed new authorship shown in section 4 **/and the addition/removal*of my name to the authorship list** /and the proposed change in corresponding author		

Please use an additional sheet if there are more than 10 authors.

In case of author collaborations with formal agreement:

	Name of consortium/consortia	First name	Family name		Signature	Date
Representative/legal guarantor				I agree to the proposed new authorship shown in section 4 **/and the addition/removal*of my name to the authorship list** /and the proposed change in corresponding author		

Both added/removed authors should complete the information in the first table under Section 6.

---- End of form ----

SPRINGER NATURE Springer Nature is one of the world's leading global research, educational and professional publishers, created in May 2015 through the combination of Nature Publishing Group, Palgrave Macmillan, Macmillan Education and Springer Science+Business Media.
